# A Case of De Novo Positional Complex Sleep Apnea Syndrome (CompSAS)

**DOI:** 10.3390/reports6030032

**Published:** 2023-07-18

**Authors:** Abdelkarim Khalifa, Marc Spielmanns

**Affiliations:** 1 Sleep Medicine Center Zurich Oberland, GZO Wetzikon, 8620 Wetzikon, Switzerland; 2 Faculty of Health, Department for Pulmonary Medicine, University Witten Herdecke, 58455 Witten, Germany

**Keywords:** central sleep apnea, adaptive servo ventilation, oxygen therapy, sleep positional, therapy

## Abstract

Obstructive sleep apnea (OSA) is well known to often improve with non-supine positioning as opposed to supine positioning. Emerging research supports a role for sleep position management in patients with central sleep apnea (CSA) as well. We report a case of de novo complex sleep apnea syndrome (CompSAS) in a 78-year-old female, who presented after a car accident due to unclear syncope. Diagnostic polysomnography (PSG) showed moderate OSA. A CompSAS developed under automatic positive airway pressure (APAP), while 4 years of downloaded data showed good adherence. No significant benefit was reported under adaptive servo ventilation (ASV) and BiPAP-ST, while a reduction in CSA in the non-supine position was noticed. Oxygen and sleep positional therapy (SPT) were considered, resulting in a significant improvement in CSA and sleep quality. Further research on the prevalence of positional CSA is needed.

## 1. Introduction

Central sleep apnea syndrome (CSAS) is a characterized by the cessation of ventilation lasting for ≥10 s due to the transient loss of neural output to the ventilatory muscles. Central sleep apnea (CSA) occurs in less than 5% of subjects admitted to sleep clinics [1]. A distinction is made between hypercapnic CSA and non-hypercapnic CSA. Hypercapnic CSA is generally seen in neurological disorders with reduced central drive to the respiratory musculature or diminished muscle strength. Non-hypercapnic CSA occurs in connection with congestive heart failure (CHF), high altitudes, hypothyroidism, and the idiopathic form of CSA.

The majority of patients with mild to moderate obstructive sleep apnea (OSA) have more apneic events in the supine position, as compared with the non-supine position. The most commonly used definition for positional OSA stipulates that the apnea–hypopnea-index (AHI) must be at least twice as high in the supine position as compared with the non-supine positions. OSA is a more common and well-described form of sleep-disordered breathing (SDB) than CSA. Indeed, supine-related OSA is a dominant phenotype of OSA with a prevalence of 20–60% in the general population [2].

Cheyne–Stokes respiration (CSR) is a periodic pattern of waxing and waning of ventilation with episodes of hyperventilation alternating with central apnea/hypopnea. CSR occurs mostly in patients with chronic heart failure and after a stroke. Some studies report that CSR occurs more often in the supine position, probably due to changes in the functional residual capacity [3]. Positional central sleep apnea (PCSA) has been also reported in a few cases, in whom no underlying disease has been ascertained [4]. The association between positional sleep apnea (PSA) and CSA remains poorly examined.

Patients with OSA may develop central apneas under the application of positive airway pressure (PAP), especially during the first days or weeks after initiation. This phenomenon has been described as complex sleep apnea syndrome (CompSAS). However, it is of crucial importance to clearly define treatment-induced CSA and separate it from treatment-independent central apneas. CompSAS contributes often to a reduced response or adherence to continuous positive airway pressure (CPAP) and may lead to CPAP failure [5].

This is a case report of a 78-year-old female with a severe de novo positional CompSAS.

## 2. Detailed Case Description

A 78-year-old female presented to her primary doctor after a car accident due to unclear syncope in 2016.

A physical exam and basic laboratory investigation, including complete blood count, comprehensive metabolic panel, and thyroid stimulating hormone, were normal. The patient had a history of hypertension and diabetes mellitus Type 2. Also, Scheuermann’s disease had been known since childhood. She had a normal weight with a BMI of 20 kg/m^2^. The patient denied alcohol or illicit drug use. A full cardiological and neurological examination did not show pathological findings. Also, her mental health history appeared to be noncontributory. As a next step, a diagnostic polysomnography (PSG) was recommended.

The patient presented at the Sleep Medicine Center at the GZO Wetzikon in Switzerland in November 2016. Polysomnography showed a moderate OSA with an average AHI of 20/h. The Epworth sleepiness scale (ESS) showed a score of 3/24, indicating lower normal daytime sleepiness. A maintenance of wakeful test (MWT) was performed the next day and provided no evidence for excessive daytime sleepiness. There were periodic limb movements during sleep (PLMS) found with an average index of 40/h. Otherwise, no other unusual behaviors were noted. Automatic positive airway pressure (APAP) therapy with pressure (9–14 cm H_2_O) was recommended. 

The patient returned 10 months later for follow-up after using APAP. The downloaded data showed an effective treatment of her sleep apnea. Adherence data showed a regular device usage of 6 h on average per night. Therapy data showed an average residual AHI of 1.2/h. She reported a subjective benefit regarding night sweats. The next follow-up visit was in February 2018, which again showed a regular device usage and an average residual AHI of 2.0/h. The ESS showed a score of 3/24, while the patient reported no subjective benefit from the therapy. The therapy data from the next follow-up in September 2019 showed a slight elevated AHI of 9.6/h in spite of the regular usage without significant air leaks. The patient denied the occurrence of excessive daytime sleepiness as well as sleep disturbances. The APAP therapy with pressure (9–14 cm H_2_O) was continued without any change. 

In March 2021, the downloaded data showed ineffective treatment of her sleep apnea with an average residual AHI of 40/h. The patient returned for an overnight titration study at the sleep laboratory. APAP pressures (9–14 cm H_2_O) were tested. This study showed a CompSAS with an average residual AHI of 32.1/h. The respiratory events were central with an average central AHI of 32.1/h. Also, the transcutaneous capnography registered a mild hypercapnia with an average transcutaneous CO_2_ pressure (PtcCO_2_) of 53.1 mmHg and maximum PtcCO_2_ of 73.8 mmHg, probably due to Scheuermann’s disease with kyphoscoliosis (Table 1). According to these findings and also in view of the patient’s clinical condition, we considered a change to adaptive servo-ventilation (ASV) therapy. Echocardiography showed a normal left ventricular ejection fraction (LVEF) of 64%. 

Follow-up visits were performed in March, June, and July 2021. The downloaded data of the ASV device showed an ineffective treatment with an average AHI of 18.3/h, 20.2/h, and 15.6/h, respectively. Adherence data showed regular device usage with an average of 5.5 h, 5 h, and 5.5 h of usage per night, respectively. No significant air leaks were noted using the AirTouch Mask F20. Again, the patient reported no subjective benefit from the therapy. Also, the downloaded data from October 2021 showed persistent ineffective treatment with an average residual AHI of 23/h. However, adherence data showed regular device usage with an average of 5.3 h usage per night. As a next step, we considered a device change using an ASV device from another producer. In June 2022, the patient experienced an acute myocardial infarction. Echocardiography showed a reduced LVEF (44%). Therefore, we stopped the ASV therapy according to the recommendation of the American Academy of Sleep Medicine (AASM). 

The patient returned for an overnight titration study with capnography. Next, a bilevel positive airway pressure-spontaneous/timed (BiPAP-ST) of 16/8 cm H_2_O was tested. A mean oxygen saturation of 94%, a minimum oxygen saturation of 83%, and an average AHI of 19.3/h were observed at this pressure setting. The transcutaneous capnography registered a normocapnia with an average PtcCO_2_ of 38.7 mmHg and maximum PtcCO_2_ of 46 mmHg.

**Figure 1:**
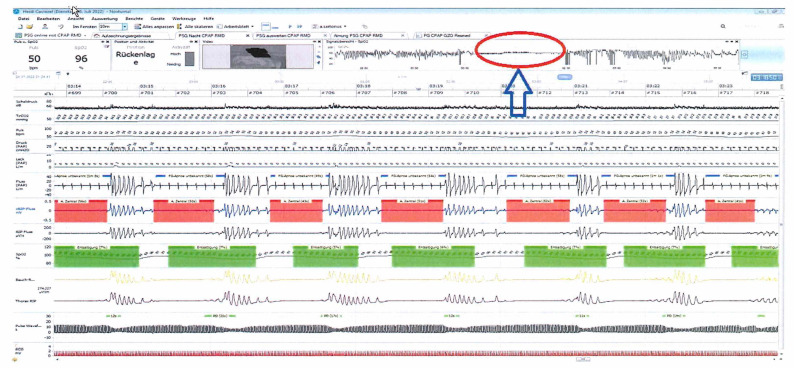
Central apneas during supine sleep on BiPAP-ST (spontaneous/timed). The central apneas are marked in red while the oxygen desaturations are marked in green. The arrow also refers to the significant improvement of oxygen desaturations during the non-supine position in the overview of the oxygen saturation during the whole study.

Since the respiratory events were predominantly central events in the supine position (Figure 1), we considered the initiation of sleep positional therapy (SPT) accompanied by nocturnal oxygen therapy with a flow rate of 2 L/minute.

**Figure 2:**
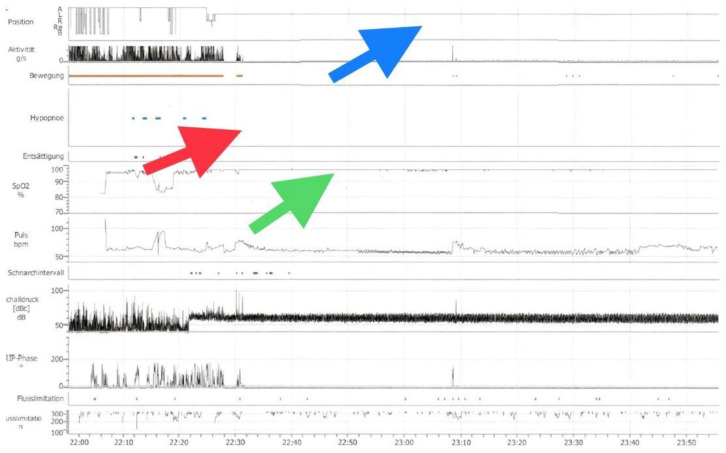
Ambulatory nocturnal polygraphy of the sleep positional therapy (SPT) and oxygen therapy. The figure above shows the improvement of the oxygen desaturations (green arrow) and hypopneas (red arrow) during sleep on the left side (blue arrow).

The ambulatory nocturnal polygraphy on SPT and oxygen therapy showed an effective treatment of the respiratory events with an average AHI of 0/h and mean oxygen saturation of 97.8% (Figure 2). The patient also reported a significant improvement of her sleep quality. 

## 3. Discussion

Sleeping position could be an aggravating factor for sleep apnea. Specifically, sleeping in the supine position can lead to a significant worsening of OSA, which is believed to be related to the relaxation of muscles in the jaw and throat under the influence of gravity, leading to the narrowing of the upper airways. This is frequently seen in patients with less severe OSA and smaller neck circumference. 

In the literature, there are only a few reports of PCSA in patients without cardiac history or CHF. The change in the pattern of the sleep-disordered breathing (SDB) from obstructive to central or mixed respiratory events with the positional change to a supine one has already been described in a study which included eight patients in whom no underlying cardiac disease was known. Two patients already had a history of cerebrovascular events (one with cerebral hemorrhage and the other with cerebral ischemia) [6]. A significant worsening of the treatment-emergent CSA/CompSAS while employing the CPAP/ASV in supine sleep has also been reported in another study [7]. Zaharna et al. also reported a case of idiopathic CSA in an otherwise healthy young man with significant worsening in supine sleep [4]. 

Benosit L. et al. published a prospective multicenter trial of 16 patients, in whom 4 patients suffered from hypertension and arrhythmia, which showed that SPT was effective and can be considered a new treatment modality in PCSA. Long-term follow-up and compliance monitoring is ongoing [8]. 

This case report showed a CompSAS that was significantly worse in the supine position. While ASV and BiPAP-ST are the first choice in such cases, no significant improvement in residual AHI was seen in this case. Therefore, we have considered SPT using a sleep pillow. An additional therapy with oxygen was recommended. The ambulatory nocturnal polygraphy after nearly 6 weeks showed a significant improvement of the average AHI without significant oxygen desaturations. The patient reported a better sleep quality without any excessive daytime sleepiness. 

Treatment of CSA is generally based on the underlying cause. In our case, we assumed that the central apneas were not due to CHF, even with a mildly reduced LVEF of 44% after the myocardial infarction in June 2022. However, CSAs were reported already in APAP overnight titration in 2021 followed by echocardiography showing a normal LVEF of 64% before the beginning of ASV therapy. Additionally, a CSR pattern and periodic breathing were absent on both the diagnostic and titration studies. Also, the patient did not tolerate opioids and was receiving nonsteroidal anti-inflammatory drugs (NSAIDs) for her chronic pain. 

The individual response of the ventilatory system to changes in the CO_2_ level can be described as loop gain. This represents the ratio of the ventilatory response to any breathing disturbances. Stimulation of ventilation by arousals increases tidal volumes or breathing frequency and reduces the actual PaCO_2_. This executive part of the loop gain represents the plant gain. High loop gain results in a ventilatory overshoot with hyperventilation and hypocapnia leading to apneas and hypoxemia. 

The peripheral and central chemoreceptors measure increases or decreases in PaCO_2_ and function as the feedback gain in a biological system. Cardiovascular failure may be associated with increased circulation time, which delays the perception of the metabolic variations at the chemoreceptors. 

Sleeping in the supine position reduces cardiac output and functional residual capacity, which consequently enhances plant gain [9]. 

Although CSA is usually associated with hypocapnia, it is not compulsory. This patient’s PtcCO_2_ was slightly elevated (mean 53.1 mmHg) in the capnography measurement during the overnight titration study employing APAP therapy in March 2021 and was normal (mean 38.1 mmHg) during the BiPAP-ST overnight titration study in July 2022. The proximity of the central apnea threshold to the carbon dioxide (CO_2_) level was more important. The slightly elevated PtcCO_2_ corresponds to the fact that hypoventilation is the predominant SDB in kyphoscoliosis patients. Eupneic patients with CHF may also have CSA. 

The prevalence of CompSAS seems to vary among different studies, ranging between 18% and 56%. However, it is not easy to assume the prevalence of CompSAS in a clinical setting, because of the dynamic nature of this condition, with improvement or disappearance of the central respiratory events during sleep in some patients and its de novo appearance in others. CompSAS may occur de novo in 4% of patients with OSA under CPAP during follow-up [10]. Only those central apneas that do not disappear under CPAP fulfill the criteria of treatment-induced CSA (CompSAS).

We assume that our patient represented a case of de novo positional CompSAS, which improved significantly with nocturnal oxygen therapy (NOT) and SPT. NOT is still indicated for CSA-CSR. While the exact mode of action is unknown, it is believed that supplemental oxygen dampens the respiratory drive, thus reducing the minute ventilation and increasing partial pressure of carbon dioxide. However, the possibility of coexisting obstructive respiratory events in the overnight titrations studies under APAP and BiPAP-ST cannot be ruled out due to the absence of esophageal pressure, which is seen as a gold standard in identification of obstructive and central respiratory events during sleep [11]. Other causes for worsening of OSA like reduced adherence, significant air leaks, and weight gain had already been excluded.

## 4. Conclusions

The present case suggests that SPT should be taken into consideration as a treatment option of CSA or CompSAS, especially in those patients who have difficulties in tolerating other treatment options for CSA including CPAP, BiPAP, and ASV therapy. In some cases, patients view SPT as a more tolerable alternative treatment. However, high scientific evidence for SPT as viable treatment option for CSA has been lacking until now.

## 5. Patents

This research was performed in the Sleep Medicine Center (GZO Wetzikon) in Zurich Oberland, Switzerland.

## Figures and Tables

**Table 1:** Results of the PSG from the APAP therapy (9–14 cm H_2_O) in the sleep laboratory.

APAP Therapy	Time
Total Recording Time	466 min
Total Sleep Time	428 min
Sleep Efficiency	91.1%
Sleep Latency	0.9 min
REM Latency	65 min
REM Periods	4
N1	2.6%
N2	47.8%
N3	25.9%
REM	15.7%
Supine	428 min
Prone	0 min
Left	0 min
Right	0 min
AHI	32.1/h
Central AHI	32.1/h (Supine 32.1/h)
Obstructive AHI	0/h
Mean Sleep Oxygen Saturation	93.4%
Minimum Sleep Oxygen Saturation	77%
Transcutaneous CO_2_ Mean	53.1 mmHg
Transcutaneous CO_2_ Maximum	73.8 mmHg

PSG: polysomnography, APAP: automatic positive airway pressure, AHI: apnea–hypopnea index, CO_2_: carbon dioxide.

## Data Availability

The data presented in this study are available in this article.

## Abbreviations

AASM	American Academy of Sleep Medicine
AHI	Apnea–hypopnea index
APAP	Automatic positive airway pressure
ASV	Adaptive servo-ventilation
BiPAP-ST	Bilevel positive airway pressure-spontaneous/timed
CHF	Congestive heart failure
CO_2_	Carbon dioxide
CompSAS	Complex sleep apnea syndrome
CPAP	Continuous positive airway pressure
CSA	Central sleep apnea
CSAS	Central sleep apnea syndrome
CSR	Cheyne–Stokes respiration
ESS	Epworth sleepiness scale
LVEF	Left ventricular ejection fraction
MWT	Maintenance of wakeful test
NSAIDs	Nonsteroidal anti-inflammatory drugs
NOT	Nocturnal oxygen therapy
OSA	Obstructive sleep apnea
PAP	Positive airway pressure
PaCO_2_	Carbon dioxide tension
PCSA	Positional central sleep apneas
PLMS	Periodic limb movements during sleep
PSA	Positional sleep apnea
PSG	Polysomnography
PtcCO_2_	Transcutaneous CO_2_ pressure
SBD	Sleep-disordered breathing
SPT	Sleep positional therapy
